# Seasonal analysis of indoor and outdoor ratios of PM_2.5_ and PM_10_ in Bangkok and Chiang Mai: A comparative study of haze and non-haze episodes

**DOI:** 10.1016/j.heliyon.2025.e42261

**Published:** 2025-01-24

**Authors:** Wissanupong Kliengchuay, Sarima Niampradit, Narut Sahanavin, William Mueller, Susanne Steinle, Miranda Loh, Helinor Jane Johnston, Sotiris Vardoulakis, San Suwanmanee, Walaiporn Phonphan, John W. Cherrie, Kraichat Tantrakarnapa

**Affiliations:** a Faculty of Tropical Medicine, Mahidol University, Bangkok, Thailand; b Faculty of Physical Education, Srinakharinwirot University, Nakhon Nayok, Thailand; c Institute of Occupational Medicine, Edinburgh, UK; d Nano Safety Research Group, School of Engineering and Physical Sciences, Heriot Watt University, Edinburgh, UK; e The Australian National University, Canberra, Australia; f Faculty of Public Health, Mahidol University, Bangkok, Thailand; g Faculty of Science and Technology, Suan Sunandha Rajabhat University, Bangkok, Thailand

**Keywords:** PM_2.5_, Indoor/Outdoor (I/O) ratios, Haze episode, Non-haze episode

## Abstract

Particulate matter (PM) air pollution has been identified as one cause of human health impact with the estimated global 8.8 million attributable deaths. Thailand experiences haze episode every year, which can lead to high ambient concentrations of particulate matter in ambient air. This study aims to investigate the relationship of indoor and outdoor air quality in both haze and non-haze period in two cities in Thailand: namely Bangkok and Chiang Mai. We conducted the air quality sampling in various styles of house, with 17 houses in both urban and rural areas, between April to October 2019. The results indicated that the concentration of PM_2.5_ in indoor air in Bangkok were 19.85 and 11.40 μg/m^3^ for haze and non-haze period, respectively, whereas the PM_10_ concentrations were 32.124 and 17.49 μg/m^3^ for haze and non-haze period, respectively. The corresponding average of outdoor air concentrations were 26.26 and 16.68 μg/m^3^ for haze and non-haze, respectively. While the PM_10_ concentrations were 46.36 and 23.86 μg/m^3^ for haze and non-haze period, respectively. In Chiang Mai, it was observed that the mean concentration of PM_2.5_ in indoor was 106.80 μg/m^3^ and 5.52 μg/m^3^ for haze and non-haze periods, respectively. Regarding PM_10_, it was observed that the mean concentration in indoor was 118.54 μg/m^3^ and 9.74 μg/m^3^ for haze and non-haze periods, respectively. Indoor/Outdoor (I/O) ratios of PM_2.5_ varied in Bangkok average was 0.76 for haze and 0.68 for non-haze period. The I/O ratio in Chiang Mai was 0.91 and 1.16 for haze and non-haze episode, respectively. Indoor/Outdoor (I/O) ratios of PM_10_ varied in Bangkok average was 0.70 for haze and 0.73 for non-haze period. The I/O ratio in Chiang Mai was 0.92 and 0.96 for haze and non-haze episode, respectively Our findings indicated the influences of outdoor air quality on indoor air quality during both haze and non-haze episode. The intrusion of outdoor air in Chiang Mai to the houses caused a higher I/O ratio than Bangkok due to the characteristics of house and culture. The indoor air quality in terms of particulate matter were dominated by outdoor air quality. Thus, people should close doors/windows during the haze as well as non-haze episode to avoiding the pollutant accumulation.

## 1 Introduction

Air pollution is a major problem that impacts on human health and quality of life [1,2]. Outdoor airborne particulate matter (PM) is a worldwide concern due to its associations with climate change and impacts on acute or chronic human health. In much of Southeast Asia pollution from industry and road transport is added to by seasonal burning of crop residue and other biomass, producing a haze which includes high concentrations of PM. Mueller and colleagues estimated that long-term exposure to PM_2.5_ has resulted in nearly 50,000 annual deaths in Thailand [3].

High concentrations of air pollutants indoors can cause acute and chronic health effects [4], particularly because people spend most time indoors [5]. Outdoor PM_2.5_ enters indoor spaces through ventilating air [6] and this adds to indoor emissions originating from household activities such as cleaning and cooking, combustion activities (including the burning of candles, use of fireplaces, use of unvented space heaters or kerosene heaters, cigarette smoking), and some hobbies [7]. However, since humans spend more than 80 % of their time indoors, contact with PM_2.5_ mainly occurs in indoor environments [8,9]. Currently, only a small number of studies have compared pollution in indoor and outdoor spaces in Thailand, but the relationship between these measures is important in understanding the potential impacts of air pollutants.

The present study aimed to assess household PM_10_ and PM_2.5_ in Thailand to compare indoor and outdoor concentrations during haze and the non-haze periods. This research provides useful information for health risk assessment and the development mitigation strategies for indoor and outdoor particulate pollution.

## 2 Materials and methods

### 2.1 Study area, study period and demographics

**Fig. 1:**
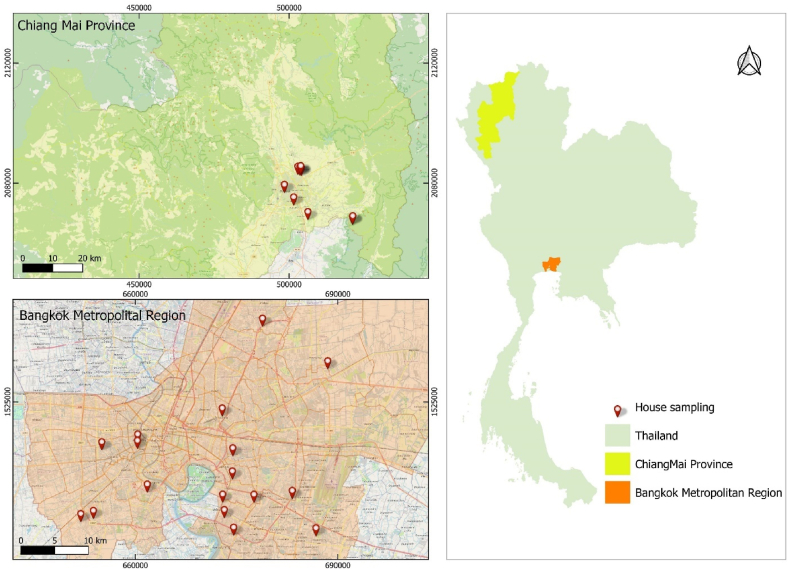
Map of the study area with house sampling sites (QGIS 3.22 Biatowieza).

This study was located in Bangkok metropolitan and Chiang Mai province of Thailand as illustration in Fig. 1. Bangkok metropolitan covers an area of 7761.6 km^2^ and has a population of over 5.6 million. Chiang Mai province includes an area of 20,107.8 km^2^ and has a population of over 1.7 million. The study was conducted between haze episode in February to March (Dry season) and non-haze episode in September to October (Rainy season) 2019 by using an opportunistic sample of homes and other indoor spaces. Ethical approval was granted from the Assessing the faculty of Tropical Medicine, Mahidol University (MUTM2018-050-01).

**Fig. 2:**
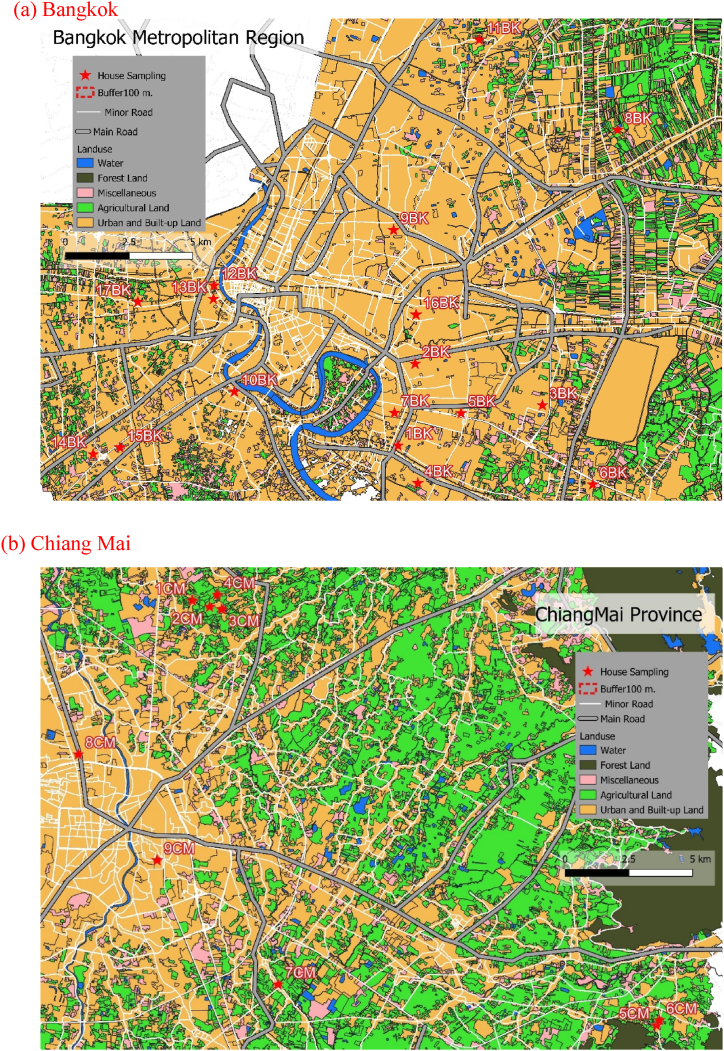
Land-use characteristics in sampling sites area (QGIS 3.22 Biatowieza).

Fig. 2 shown the land use are of sample divided into the main road, water, forest land, miscellaneous, agricultural land and urban and built-up land link with sample house [10]. Wind speeds and directions provides a detailed understanding of the prevailing wind patterns in sampling month present in Fig.SS1. The monitoring in Bangkok were measured in March the winds are stronger and predominantly from the south (average 2.26 m/s) and October as winds are weaker and distributed between south and northwest (average 1.15 m/s). In Chiang Mai were measured winds come from the north (N) direction in February (0.27 m/s).

### 2.2 Particulate matter measurements

Monitoring was carried out using a light scattering Portable Particulate Monitor (AQ-S500) (www.aeroqual.com), to assess PM_2.5_ and PM_10_. The AQ-S500 was housed in a sampling enclosure, the light-scattering optical particle sensor, with RH correction, measures particulate matter levels ranging from 0 to 1000 μg/m^3^. The measurement equipment was validated by co-location with gravimetric methods as illustrated in Fig.SS2. The PM_2.5_ and PM_10_ concentrations showed a strong correlation, with correlation coefficients (r) of 0.67 and 0.57, respectively. This indicated a consistent trend in the same direction for both PM_2.5_ and PM_10_. In addition, this method can be used in accordance with the announcement of the Pollution Control Department, regarding the general measurement and measurement methods for the average value of gas or particulate matter in the atmosphere. Moreover, the equipment was also compared with the Gravimetric method, the correlation coefficients (r) was high as 0.97. The AQ-S500 sensor was housed in a sampling enclosure and mounted on a pole at a height of 1.0–1.5 m, and was 1 m left from walls, doors and air conditioning, both indoors and outdoors [11].

The monitors were placed at the foyer of the home (indoor) and terrace (outdoor). Indoor and outdoor measurements were made simultaneously over 24 h at each house. The monitoring equipment was put inside and outside house. The Aeroqual monitor was in the living room and outdoor it was in the balcony. The samplers were placed about 1.5 m off the ground (breathing level) and at least 1 m away from anything around it. Outside, the Aeroqual monitor was set in a box and placed 1.5–2 m high on the balcony, at least 1 m away from the building (Fig.SS3). PM_2.5_ and PM_10_ data were recorded be Aeroqual monitors were set to record concentrations every 5 min throughout on 24 h. The data measurements were stored on the device and downloaded to a computer via bundled software and a USB cable at the end of the day. The filtered data is presented in Fig.SS4. However, the data underwent a filtering process to concentrate the analysis on the ratio of outdoor pollutants and the influence of indoor sources. This process was similar to the approach from Ref. [12]).

### 2.3 Data analysis

All statistical analyses were made in XLSTAT Statistical Software with data processing. Descriptive statistics were used to summarize the data; mean, standard deviations, min, and max, percentiles were used to illustrate data as appropriate. Linear regression was used to explore the relationship between outdoor and indoor PM_2.5_ concentrations. Python was used to compare cumulative distribution functions (cdf) with normal, lognormal and gamma distributions. R-Software (Openair Package) for simulating and analyzing wind patterns based on data derived from Bangkok and Chiang Mai provinces.

The I/O ratio is a straightforward and valuable parameter for qualitatively assessing the impact of outdoor sources on indoor PM levels [13]. Ratios of indoor to outdoor I/O PM were calculated using the arithmetic averages of 1-h concurrent measurements. The I/O ratio is the most used indicator for characterizing indoor air quality and the contribution of outdoor pollutants inside buildings [14]; [15,16]. To calculate the I/O ratio, Eq. (1) was used. The I/O ratio was employed to determine the relationship between indoor and outdoor pollution. This study explored the implications of the I/O ratio on indoor air pollution as defined in Eq. (1):

(1) $$I/O=C_{in}/C_{out}$$

where C_in_ and C_out_ are the indoor and outdoor PM_2.5_ concentration, respectively.

## 3 Result and discussion

### 3.1 House microenvironment

The data were collected from 17 houses in Bangkok (BKK) and 9 houses in Chiang Mai (CM). Table 1 show the descriptive information covering various aspects of the home microenvironments included in the study. The characteristics included in the table cover the number of persons at each site, the distance to the nearest road, the usage type, the type of building, and environmental factors such as smoking, mosquito coil usage, window status, and air conditioning.

**Table 1:** The characteristics of the house microenvironments.

Sampling site No.	Number of persons	Distance of nearest roads (meter)	Usage	Type of building	Smoke	Mosquito coil	open window	window open hrs.	Air condition
BKK 1	1	31.42	living	Condo	no	No	Yes	8	Yes
BKK 2	2	18.93	living	Single House	no	No	Yes	12	No
BKK 3	3	143.84	living	Single House	no	No	No	0	Yes
BKK 4	4	21.15	living	Single House	Yes	No	Yes	12	No
BKK 5	4	10.54	living	Town house	no	No	Yes	10	No
BKK 6	2	217.15	living	Single House	no	No	No	0	No
BKK 7	3	21.19	living	Town house	no	Yes	Yes	8	No
BKK 8	5	14.25	living	Single House	no	No	Yes	10	No
BKK 9	3	23.68	living	Officials' Residence	no	No	Yes	8	No
BKK 10	5	40.27	living	Officials' Residence	no	Yes	Yes	8	No
BKK 11	3	15.73	living	Town house	no	No	Yes	6	No
BKK 12	2	5.18	living	Single House	no	No	Yes	10	No
BKK 13	3	34.48	living	Temple	no	No	No	0	No
BKK 14	3	81.53	living	Single House	Yes	No	No	0	Yes
BKK 15	2	5.57	living	Town house	no	No	Yes	8	No
BKK 16	3	34.41	office	School	no	No	No	0	Yes
BKK 17	3	16.43	office	Temple	no	No	No	0	Yes
CM 1	5	767.25	living	Single House	no	No	Yes	4	No
CM 2	6	1010.65	living	Single House	no	No	No	0	Yes
CM 3	5	1228.47	living	Single House	no	No	No	0	No
CM 4	4	756.32	living	Single House	no	No	No	0	Yes
CM 5	4	104.35	living	Single House	Yes	No	Yes	24	No
CM 6	5	7.40	living	Single House	no	No	No	0	Yes
CM 7	4	26.36	living	Single House	no	Yes	Yes	24	No
CM 8	4	32.35	living	Town house	no	No	Yes	10	Yes
CM 9	3	143.45	living	Town house	no	Yes	No	0	Yes

The data are from Bangkok (BKK) and Chiang Mai (CM).

In developed countries, people spend around 65 % of their daily time at home [17]. The majority of the buildings in Bangkok were single houses, followed by town houses, whereas those in Chiang Mai were mostly single houses, with a few town houses. Smoking is reported only in Bangkok and not found in Chiang Mai sample, potentially leading to better indoor air quality in Chiang Mai. Also, the mosquito coil usage was slightly higher in Chiang Mai, possibly due to a higher presence of mosquitoes or different personal use patterns. Ventilation at both locations showed a strong reliance on natural ventilation from open windows, with Chiang Mai having longer window open hours. Air conditioning usage was similar in both locations, suggesting a balanced reliance on natural ventilation and air conditioning.

**Fig. 3:**
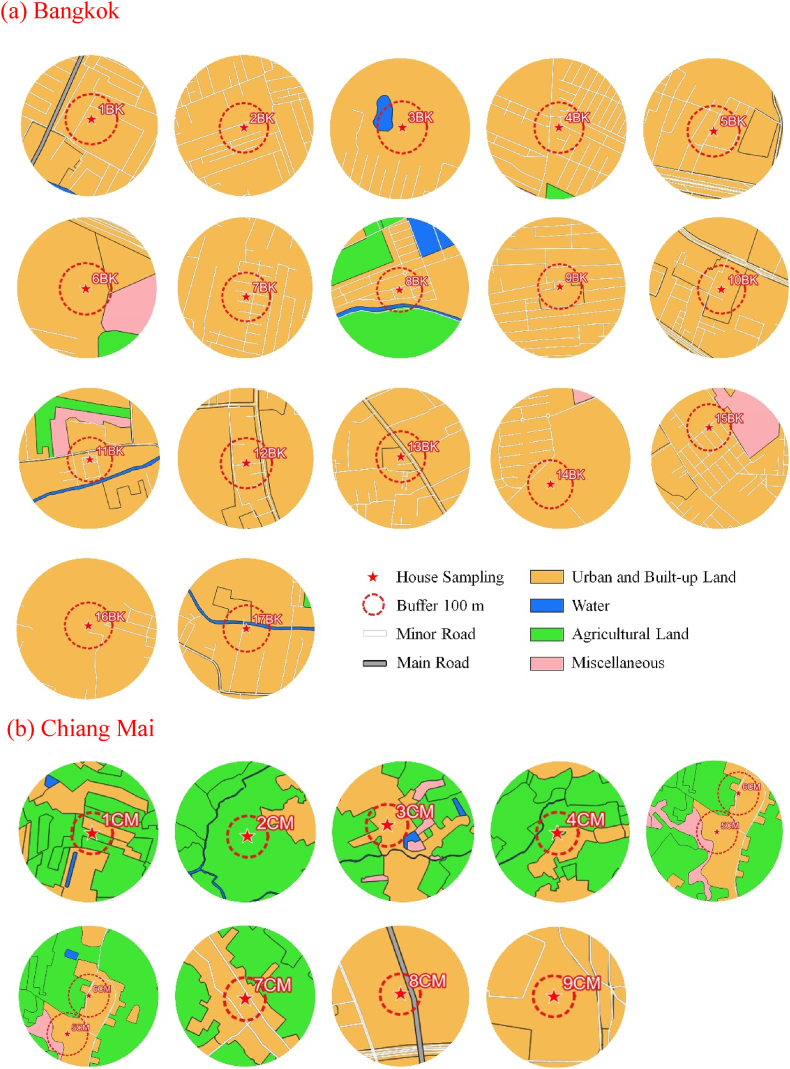
Representative land use areas for each type are marked by a circle with a 100-m radius around the house in a) Bangkok and b) Chiang Mai.

The land use area with a 100-m radius (Table SS1.) around the Bangkok and Chiang Mai house has present in Fig. 3. In Fig. 3a present the distribution of land use types reflects a mix of urban and built-up areas, covering 378,242.25 m^2^, with 17 houses under this category and all sample house are connect main road or local road of Bangkok. Agricultural land and water bodies encompass approximately 11,782.60 m^2^ in Chiang Mai house (Fig. 3b), the landscape of land use types also reveals a combination of residential, agricultural, and water areas, covering 203,665.43 m^2^, 58,214.71 m^2^, and 62,633.02 m^2^, respectively.

**Fig. 4:**
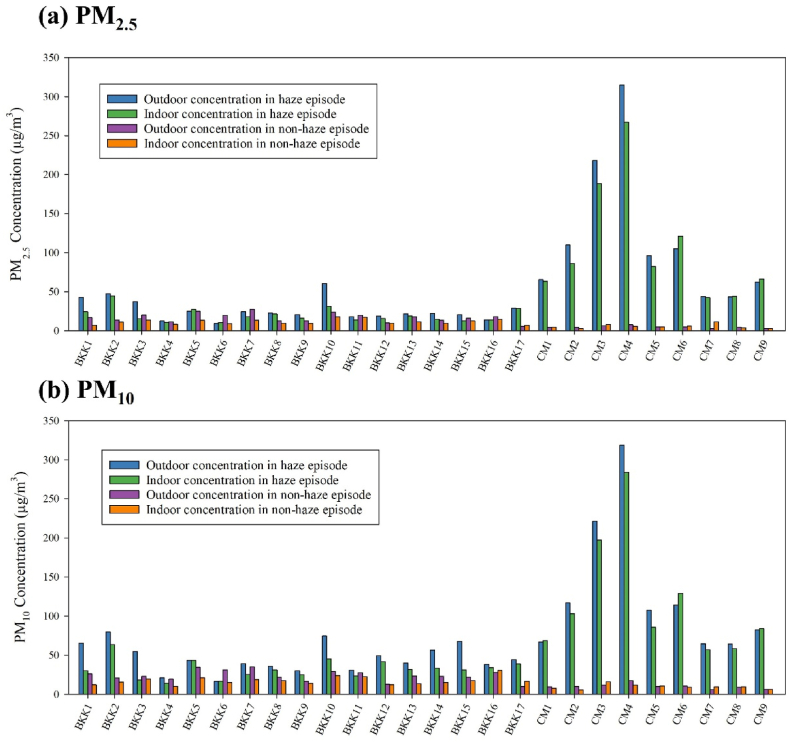
Daily concentrations of (a) PM_10_ and (b) PM_2.5_ in Haze and Non-haze episode.

Indoor and outdoor PM_10_ and PM_2.5_ concentrations (μg/m^3^) during both haze and non-haze episode are shown in Fig. 4a and b (Table SS2 and Table SS3) for each of the house, measured indoors and outdoors. The daily mean concentrations indicated that PM_10_ were higher than PM_2.5_ in all samples, with outdoor concentrations generally exceeding indoor levels. Chiang Mai samples consistently exhibited higher concentrations compared to Bangkok. Additionally, concentrations during the haze episode were higher than those in the non-haze episode across all locations. Notably, there were larger changes in indoor versus outdoor concentrations in Bangkok houses for both haze and non-haze situations, compared to the more uniform trend observed in Chiang Mai. In Chiang Mai houses were presented a consistent and steep rise in concentrations. During non-haze conditions, Chiang Mai had lower average concentration of PM_10_ and PM_2.5_ compared to Bangkok but experienced significantly higher concentrations of these pollutants during the haze episode.

### 3.2 Comparison of I/O ratios of particulate matter in selected homes

**Fig. 5:**
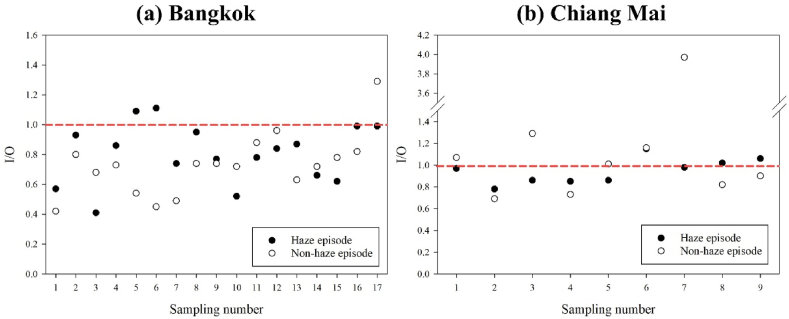
I/O ratio of PM_2.5_ in (a) Bangkok house and (b) Ching Mai house in haze and non-haze episode.

I/O ratios are shown in Table SS4. for both episodes, there are relatively consistent data with the value of indoor/outdoor (I/O) ratios of PM_2.5_ and PM_10_ in Bangkok houses and Chiang Mai houses. In Bangkok, during the haze episode, approximately 88 % of the I/O ratios for PM_10_ and 94 % for PM_2.5_ were less than 1.0 (Fig. 5a). This suggests that outdoor PM_10_ and PM_2.5_ concentrations were higher than indoor levels in general of cases during the haze period. In contrast, in Chiang Mai, the I/O ratios for both PM_10_ and PM_2.5_ were less than 1 in 67 % of sample houses during the haze episode. This indicates that outdoor concentrations exceeded indoor levels in two-thirds of the measurements in Chiang Mai. However, in the non-haze episode, the percentage of I/O ratios less than 1 decreased to 56 % for Chiang Mai. This implies that the influence of outdoor sources on indoor air was lower during the non-haze episode compared to the haze period. The remaining 33 % and 44 % of I/O ratios in Chiang Mai during the haze and non-haze episode, respectively, were greater than or equal to 1. This suggests that indoor sources or activities contributed significantly to the indoor PM concentrations in this study (Fig. 5b).

The comparison of I/O ratio for PM_2.5_ concentrations with those of other cities is presented in Table SS5. However, this instruction holds true only for buildings with natural ventilation or mechanical ventilation systems that do not involve air refreshing or particle filtration mechanisms [14]. We found that the I/O ratios of both PM_10_ and PM_2.5_ in both haze and non-haze are higher than 1 in many houses in Chiang Mai. That means there are some emission sources of both PM in their houses.

These studies suggested that I/O ratios can vary considerably depending on various factors, for example, outdoor concentrations, building characteristics, locations of the house, equipment and type of building [11,18]. One influencing factor of both ambient air quality and indoor air quality is the burning of fossil fuel, the promotion of renewable biofuel would be benefits in terms of reducing air pollution as reported in the previous studies [19]; [20]. However, as Damin suggested, cooperation among relevant parties is the key factor [21].

The findings derived from using linear regression analysis and calculated correlation coefficient, are found the strength and direction of the linear relationship between the two variables under investigation. Additionally, the coefficient of determination, represented by r-squared (R^2^), indicates the proportion of variance in the dependent variable that can be explained by the independent variable through the linear regression model shown Y = a + bX or C_in_ = a+bC_out_ without the haze and with haze episode (Fig.SS5 and Fig.SS6). PM_2.5_ concentrations C_in_ (indoor) and C_out_ (outdoor), and a and b are the intercept, and slope of linear regression, respectively [22]. The positive correlation is in Bangkok house in haze episode, non-haze episode, and haze episode in Chiang Mai had coefficients of determination (R^2^) = 0.60, 0.56, and 0.98, respectively. Whereas no relationship was found in the non-haze episode of Chiang Mai where the coefficient of determination was very low (R^2^ = 0.002.)

**Fig. 6:**
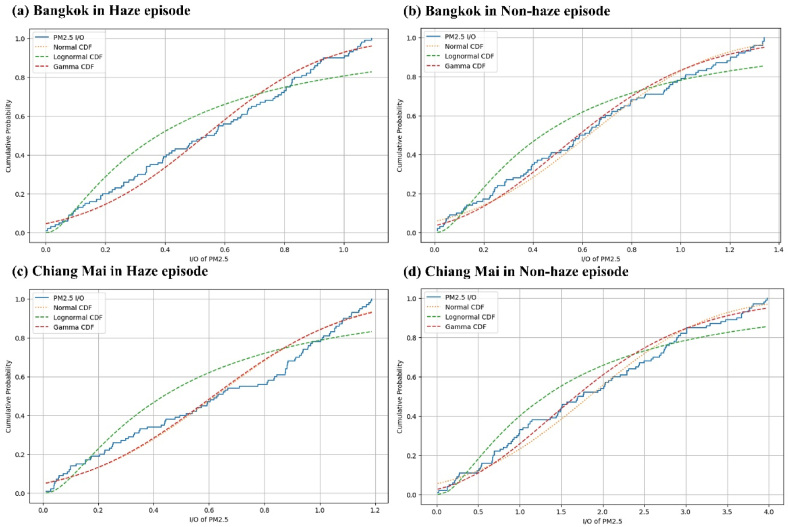
Cumulative distributions function of PM_2.5_ I/O ratios and best curve fitting in (a) Bangkok in Haze episode, (b) Bangkok in non-haze episode, (c) Chiang Mai in Haze episode and (d) Chiang Mai in non-haze episode.

Fig. 6a and b illustrates the cumulative distribution I/O ratios of PM_2.5_ in Bangkok houses. However, found that on haze and non-haze (93 % and 83 %) of these ratios are equal to or less than one, compared to Chaing Mai were found 84 % and 23 % on haze and non-haze episode as presented in Fig. 6c and d. The high I/O ratios of PM_2.5_ are frequently observed in haze episode in both provinces, accepted non-haze episode in Bangkok are likely due to the resuspension of dust caused activities and movement, which significantly increases indoor PM_2.5_ concentrations, as there is no distinct indoor source of pollution in such a microenvironment [23]. Moreover, the patterns of ventilation differ based on climate, with a higher tendency to keep windows and doors open in warmer regions as opposed to cooler ones, and more generally during the summer months rather than in winter [17]. According with the reported by Massey, who suggests that the houses utilize natural ventilation, it is important to further explore the relationship between indoor and outdoor air quality data [24].

## 4 Conclusion

This study showed that outdoor particulate air pollution is related to haze episode, which significantly increased the indoor air pollutant concentrations. Measurements of PM_10_ and PM_2.5_ concentrations were conducted during the non-haze and haze episode in both provinces. The average I/O ratios of PM_10_ were lower than the I/O ratios of PM_2.5_, The indoor and outdoor PM_2.5_ concentrations show a significant positive correlation; for Bangkok in both episode and haze episode in Chiang Mai, with the coefficient of determination (r^2^ = 0.60, 0.56, and 0.98), respectively. This indicates that the indoor PM_2.5_ concentrations were dominated by outdoor pollution. However, the low correlation coefficient of PM_2.5_ concentrations indoors and outdoors in Chiang Mai during the non-haze episode illustrates the effectiveness of rainy episode reducing outdoor pollution in this region. The findings from this study reveal that during non-haze there are emission sources of particulate matter both sizes in their houses, there should be some campaigns to reduce the indoor particulate matters sources. Regarding analytical approximations of pdf/cdf, it has been noted that the PM_2.5_ I/O ratios for two episodes of Bangkok house and Chiang Mai in haze microenvironments are more effectively exhibited by a normal distribution function rather than by gamma or lognormal distribution functions, differing non-haze in Ching Mai.

The studies using the technique investigated in this research open up novel avenues for mitigating particulate matter (PM) concentrations, effectively decreasing PM levels. This approach holds the potential to substantially enhance indoor air quality, thereby promoting improved environmental health conducive to the well-being of people. The implications of this methodology extend far beyond merely reducing airborne contaminants; it paves the way for creating indoor air that nurtures and protects human health, ultimately promoting improved efficiency and overall quality of life.

## CRediT authorship contribution statement

**Wissanupong Kliengchuay:** Writing – original draft, Methodology, Formal analysis, Data curation. **Sarima Niampradit:** Writing – original draft, Formal analysis. **Narut Sahanavin:** Writing – original draft, Formal analysis. **William Mueller:** Writing – review & editing, Writing – original draft, Conceptualization. **Susanne Steinle:** Writing – original draft, Methodology, Formal analysis. **Miranda Loh:** Writing – review & editing. **Helinor Jane Johnston:** Writing – review & editing, Conceptualization. **Sotiris Vardoulakis:** Writing – review & editing. **San Suwanmanee:** Writing – review & editing, Writing – original draft. **Walaiporn Phonphan:** Writing – original draft, Formal analysis. **John W. Cherrie:** Writing – review & editing, Visualization, Supervision, Conceptualization. **Kraichat Tantrakarnapa:** Writing – review & editing, Supervision, Project administration, Funding acquisition, Conceptualization.

## Data availability

Data used and analyzed in the manuscript will be made available upon request to the corresponding author.

## Declaration of competing interest

The authors declare the following financial interests/personal relationships which may be considered as potential competing interests: Kraichat Tantrakarnapa reports financial support was provided by Thailand Research Fund (10.13039/501100004396TRF) (RDG603009). Kraichat Tantrakarnapa reports financial support was provided by 10.13039/501100004704National Research Council of Thailand (10.13039/501100004704NRCT). John Cherrie reports financial support was provided by 10.13039/501100000265Medical Research Council (MRC). Reports a relationship with that includes:. Has patent pending to. If there are other authors, they declare that they have no known competing financial interests or personal relationships that could have appeared to influence the work reported in this paper.
